# Slow-release non-protein nitrogen sources in animal nutrition: A review

**DOI:** 10.1016/j.heliyon.2024.e33752

**Published:** 2024-06-27

**Authors:** Masoumeh Niazifar, Maghsoud Besharati, Muhammad Jabbar, Shakira Ghazanfar, Muhammad Asad, Valiollah Palangi, Hüseyin Eseceli, Maximilian Lackner

**Affiliations:** a Department of Animal Science, Ahar Faculty of Agriculture and Ntural Resources, University of Tabriz, Iran; b Faculty of Biosciences, Department of Zoology, Cholistan University of Veterinary and Animal Sciences Bahawalpur, Pakistan; c National Institute of Genomics and Advanced Biotechnology, Pakistan Agricultural Research Council Islamabad, Pakistan; d Department of Zoology, Division of Science and Technology, University of Education Lahore, Punjab, Pakistan; e Department of Animal Science, Faculty of Agriculture, Ege University, 35100, Izmir, Turkiye; f Department of Nutrition Sciences, Faculty of Health Sciences, Bandirma Onyedi Eylul University, TR, 10200, Bandirma, Balikesir, Turkiye; g Department of Industrial Engineering, University of Applied Sciences Technikum Wien, Hoechstaedtplatz 6, 1200, Vienna, Austria

**Keywords:** Non-protein nitrogen (NPN) sources, SRU (slow-release urea), Biuret, Nitrogen, Protein, SCP (single cell protein)

## Abstract

Today, feeding protein supply according to need in high-yielding lactating cows has become a big challenge. Protein is the most costly bulk constituent of animal diet, and the price of protein sources is increasing steadily, which is different from milk price rising. Therefore, one way for farmers to reduce feed costs is to reduce dietary protein share. Ruminants obtain their amino acids from 2 sources: amino acids from ruminally undegraded protein (RUP) and microbial protein synthesized in the rumen. A key goal in ruminant nutrition strategies, maximizing the use of rumen degradable protein (RDP), is through its efficient conversion into microbial protein. Urea is a supplement and a possible source of non-protein nitrogen (NPN) in ruminants' diets which meets bacteria's ammonia needs. Rumen ammonia sources include protein, peptides, amino acids, and other nitrogen-bearing compounds. As urea, uric acid, nitrate, and possibly nucleic acid are rapidly converted to ammonia, the ammonia reservoir indicates that the ruminal metabolism of ammonia is relatively small. Bacteria in the rumen can obtain between 40 and 95 percent of their nitrogen demand from ammonia, depending on their diet. Using NPN (non-protein nitrogen) as a reliable nitrogen source for ruminants was recognized over 100 years ago. Urea is quickly released in the rumen, its use in the diet is limited due to ammonia toxicity. So, the solution to this problem is that the product in nitrogen release rate from urea changes according to the digestion of fibers in the rumen. In the past, several slow-release products were made and evaluated. Slow-release urea (SRU) sources will also affect microbial growth and livestock performance compared to conventional plant protein sources. Acceptance of SRU sources, depending on their price compared to conventional plant protein ingredients is feasible. Studies has shown that the use of slow-release urea did not have a negative effect on digestibility, rumen parameters, milk production and livestock performance. Single-cell protein (SCP) is an emerging alternative protein source, currently being mainly studied for chicken and aquatic species.Finally, it is concluded that slow release urea can be used in feeding ruminants without any side effects.

## 1 Introduction

Nitrogen is an expensive ingredient in feed, yet it is not used efficiently. In the Netherlands, for instance, 29 % of nitrogen in livestock feed ends up in the product (meat, eggs, milk), while most of it is wasted on manure [1].

Ruminants obtain their amino acids from 2 sources: amino acids from ruminally undegraded protein (RUP) and microbial protein synthesized in the rumen [2]. In the next 50 years, global meat and milk production must double proportionate to population growth and increasing wealth development, especially in developing economies. In counturies with limited water resources, and precipitation, lack of optimal use of available facilities, and required mechanization have caused a significant shortage of animal feed resources. Therefore, animal feed costs in these country are significantly higher than the global average [3]. There are many problems with ruminant feed due to the growing human need for cereals, more attention has been paid to increasing forage productivity in ruminants' diets. In the case of optimal rumen microbe growth, rumen fermentation can provide 70–100 % of the required protein by ruminants. In addition, 70–85 % of the energy source can be assimilated as VFA (volatile fatty acids) [4]. A key goal in ruminant nutrition strategies, maximizing the use of rumen degradable protein (RDP), is through its efficient conversion into microbial protein [5]. Urea (carbamide, CO(NH_2_)_2_) is a supplement and a possible source of non-protein nitrogen (NPN) in ruminants' diets which meets bacteria's ammonia needs. Bacteria in the rumen can obtain between 40 and 95 percent of their nitrogen demand from ammonia, depending on their diet [6], although protozoa do not utilize ammonia. The economic benefits of using urea instead of traditional protein sources are the most important driving factor for utilizing urea as a nitrogen source, which is cheaper per unit of nitrogen than other nitrogen sources. In addition, transportation efforts and transportation costs are high despite various factors affecting urea transport. It is considered a dense nitrogen source, and its transportation cost is lower than that of other nitrogen sources incorporated in animal feed [7].

The use of urea in ruminant diets is economically viable, due to its low price. This is due to the availability of these resources. Even though urea is quickly released in the rumen, its use in the diet is limited due to ammonia toxicity. Furthermore, the high solubility of urea and slow rate of intra-rumen fiber digestion creates a discrepancy between the availability rate of urea-derived ammonia and the pace of nitrogen uptake by fiber-fermented bacteria. If not properly managed, the risk of urea overdose may result in increased blood ammonia levels, decreased feed intake and performance, and potentially fatal ammonia poisoning. So, the solution to this problem is that the product in nitrogen release rate from urea changes according to the digestion of fiber in the rumen. In the past, several slow-release products were produced and evaluated; these include:

"Biuret" [8], Starea [9], urea-formaldehyde [10], uromol [11], urea coated with flaxseed oil and talc [12], lactosylurea [13], polyvinyl alcohol urea [14], urea and lignocellulosic complex [15], uromalt [16], isobutyraldehyde monourea [17], Epogen [18], microcapsules [19], and calcium chloride-bonded urea [20].

Acceptance of slow-release urea (SRU) sources, depending on their price compared to conventional plant protein ingredients is feasible.It is therefore possible to increase the acceptance of this supplement by farmers by synthesizing a local product at a lower cost than a foreign imported good, which satisfies ruminant needs. The efficacy of protective measures is contingent upon the strong bonding of the urea bond and its accessibility to the rumen. However, certain products like biurets necessitate adaptations that restrict their usage [21]. Biuret (carbamoylurea, allophanamide) is condensed urea. In high-fiber diets, SRU may prove to be a beneficial supplement owing to its low rate of N-release in the animal's rumen.

## 2 The role of ammonia in ruminal fermentation

Rumen ammonia sources include protein, peptides, amino acids, and other nitrogen-soluble compounds. As urea, uric acid, nitrate, and possibly nucleic acid are rapidly converted to ammonia, the ammonia reservoir indicates that the ruminal metabolism of ammonia is relatively small. In addition, nitrogen flow is relatively fast.

**Fig. 1:**
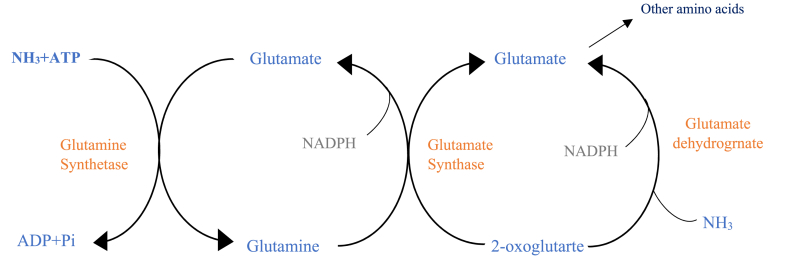
Two-step process by which ammonia is assimilated by bacteria [22,23].

Fig. 1 shows the assimimilation of ammonia-N by rumen bacteria. It is a two-step-process.Ammonia amounts entering the reservoir (rumen) by quantity and protein breakdown, or the amount and method of urea supplementation vary over a wide range. The amount of ammonia in the reservoir can change rapidly as feed is available. The maximum rate of microbial synthesis at an ammonia concentration is 5–8 mg/dL. The researchers reported the optimal ammonia concentration as a variable and believed it could vary depending on the type of diet [22]. Some studies have stated that the optimal concentration based on the type of diet consumed is 15–20 mg/dl. Maximum bacterial cell growth requires high ammonia concentrations; it is suggested that ruminal microorganisms have a similar mechanism concerning soil microbe participation. At low ammonia concentrations, the two-step cycle of bacterial growth requires glutamine synthetase and glutamate synthase [20]. These reactions depend on the conversion of glutamate to glutamine and then reducing glutamine nitrogen amide to 2-oxoglutarate using ATP [7].

## 3 Discovery and history of using urea as animal feed

**Fig. 2:**
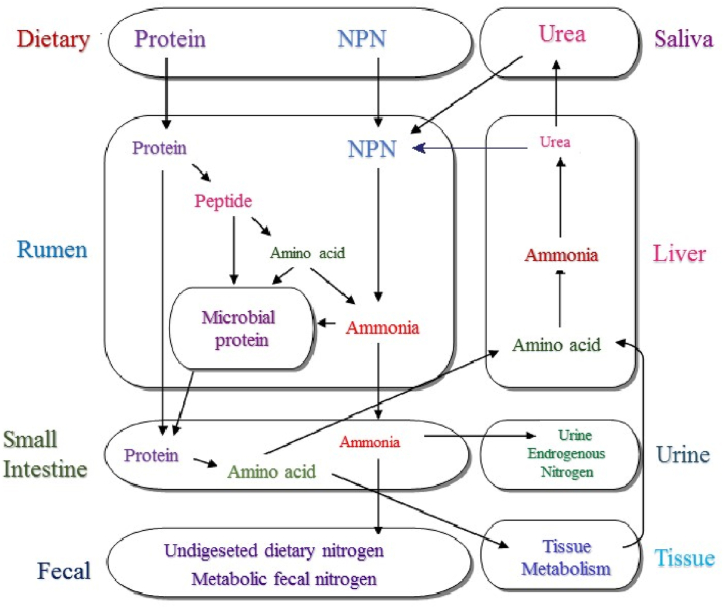
A model of the metabolism of nitrogen in the rumen [22,23].

Using NPN (non-protein nitrogen) as a reliable nitrogen source for ruminants was recognized over 100 years ago [24](Fig. 2). Already in 1891, Zuntz [25] introduced the theory that ruminant pre-gastric bacteria can utilize simple nitrogen compounds such as amides or ammonia to produce microbial protein by digesting them in the small intestine, which becomes available to livestock [24]. In 1949, Loosli et al. [26] used pure diets for ruminants, with urea being their only nitrogen source. They were the first researchers to identify ten essential amino acids, which are highly synthesized by ruminants from urea as the only nitrogen source [24]. A few years later, in a study of heifers with ruminal fistulas, Duncan et al. [27] showed that the rumen microbial population produced all the amino acids needed for ruminants in sufficient quantities. After conducting several long-term experiments on dairy cows, it was discovered that they could fulfill their maintenance and reproductive requirements and produce the typical amount of milk even when fed a protein-free diet. These findings prompted a considerable amount of research into the consumption of non-protein nitrogen and degradable proteins in the rumen. This research essentially leads to the classification of feed nitrogen as accessible to ruminal microorganisms and the part that escapes from a breakdown in the rumen and may be available through digestion in the small intestine [3].

Nitrogen metabolism in the rumen, which has been recognized as the most important factor in improving the efficiency of nitrogen consumption [28], is divided into two parts: protein that can be decomposed in the diet and microbial protein synthesis. It is divided in the stomach [29]. It is well known that decomposable protein nitrogen in the rumen and ammonia cycle in the animal body are the two main sources of nitrogen consumption by microorganisms [30]. Therefore, the amount of degradable protein is one of the most effective sources for microbial growth and improving the efficiency of nitrogen consumption (Fig. 2).

## 4 Urea

Urea was accidently discovered in 1800 by Friedrich Weller when he was making ammonium cyanate with silver cyanate. Due to its high palatability, urea is hydrolyzed rapidly into ammonia by the rumen bacteria upon entering the rumen [31]. The rapid rate of urea hydrolysis may exceed the ability of rumen bacteria to utilize ammonia in the rumen for use in microbial protein synthesis and rumen fermentation resulting in nitrogen loss [3]. Urea is the most common NPN compound in the diet of ruminants, so they use it to make microbial proteins. Urea is a cheap way to provide breakdown protein for ruminants. If natural protein can be entirely or in part derived from an NPN source, this replacement reduces feed costs.

**Fig. 3:**
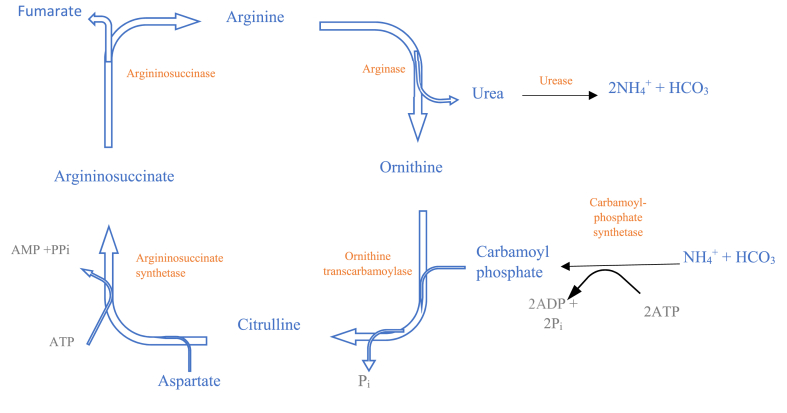
Urea cycle in the liver of ruminants [23].

Urea is converted to microbial protein by the activity of microorganisms and ruminal ammonia and provides much protein for the host animal [32]. The slow process of microbial growth and degradation in the rumen may be responsible for the rapid surge in rumen ammonia following the consumption of urea. This can result in strong nitrogen loss missing from microbial growth and protein production, as noted by Taylor-Edward [33]. The solubility of the digested protein inside the animals’ rumen is among the most crucial physical and chemical properties that impact its breakdown in this environment. Because rumen microorganisms and their enzymes must be in direct contact with proteins to break them down, usually soluble proteins that are more available to microbes and their enzymes decompose more and faster than insoluble proteins in the rumen [3]. Livestock can benefit from the conversion of non-protein nitrogen (NPN) into high-quality protein by rumen microbita. Rumen microorganism also break down high-quality, expensive dietary proteins into ammonia. The amount of urea used in the diet is relatively limited due to the rapid hydrolysis of ammonia in the rumen [34] (Fig. 3).

## 5 Strategies to minimize nitrogen loss

Nitrogen species, as summarized by Holder [24], are amongst the most relevant sources of pollution from ruminants, next to methane. Nitrogen has been especially important in dairy cows, and nitrogen pollution leads to accumulation in natural water resources, contamination of groundwater with nitrate, and atmospheric pollution with the release of nitrogen as ammonia [24]. Reducing nitrogen pollution is crucial at every stage of production, including crop cultivation, feeding, and management. Livestock consumption nitrogen efficiency can be decomposed based on the compatibility of nitrogen and non-degradable nitrogen in the diet, which is improved by livestock needs. As protein production increases, the need for storage per unit of protein production decreases. This approach seems complicated, given farmers' efforts to minimize ration costs. The most important step forward is to adapt the feed and nitrogen requirements changing the nutrition system from crude protein content due to the rumen available nitrogen and the availability of rumen undegradable protein (RUP).

The small intestine's digestive microbial protein, derived from available protein, serves as the primary source of amino acids. Overconsumption of degradable nitrogen can result in excessive nitrogen loss through urine. In contrast, its deficiency in the diet can reduce microbial protein digestion and production [32]. Over-feeding of DIP (degradable intake protein) or RUP or an imbalance between them will increase crude protein requirements for a given amount of production, resulting in more nitrogen excretion per unit of nitrogen. Diets high in crude protein, even with a balance of degradable protein and non-degradable protein, cause nitrogen to be excreted at a higher rate [3]. Nitrogen use efficiency in cows is usually relatively low under normal production conditions. Urea is very inefficient in producing protein products, and therefore, its widespread use in ruminant diets can be partly due to the low nitrogen yield in cows with low urea consumption due to faster hydrolysis of urea into the feed. Ammonia is consumed by microbial enzymes relative to the rate at which it is consumed by rumen bacteria. This causes intra-ruminal accumulation of ammonia, which is absorbed and excreted in the urine. Therefore, many efforts are made to obtain a product from urea that decomposes much slower than urea in the rumen. This is followed by ammonia participation inmicrobial metabolism and less urea excretion via urine.

## 6 Types of coatings for urea

Urea is the most frequently used source of protein supplements to meet ruminal requirements, but its rapid release may cause poisoning. To address this issue, products with a urea or N–NH_3_ emission rate similar to the rate of intra-rumen fiber digestion have been developed. Historical methods to minimize N–NH_3_ release from urea include the following techniques:

biuret [8,35]

isobutyraldehyde monourea [17]

lactosyl-urea [13]

starea [9]

urea coated with linseed oil and talc [12]

urea-corn-carboxy-resin [36]

urea-formaldehyde [10]

urea-lignocellulose complex [15,37]

urea-polyvinyl-alcohol [14].

Uromalt ® [16].

Uromol ® [11].

Table 1 summarizes the impact of slow-release urea formulations on animal performance.

**Table 1:** Influence of slow-release urea (SRU) products on nutrient digestibility and dry matter intake (DMI). Reproduced from Andrieu and Wilde [4].

Source	Type of SRU	Suppl., % diet	Animal	DMI kg/d	Digestibility, % –			
					DM	CP	DF	OM
Puga et al. [38],	Urea	0	Sheep	5.9	8.6	–	67.8	57.6
	9. Control Release	30^a^		8.2	64.8	–	74.0	63.2
Galina et al. [39],	SRU	0	Beef	5.8	58.8	–	57.1	48.4
	10. SRU	1.8		8.2	68.7	–	75.1	59.7
Highstreet et al. [34],	Urea	1.8	Cows	28.2	–	70.9	50.9	–
	11. Encapsulated urea	1.7		28.6	–	70.8	50.0	–
Xin et al. [40],	Urea	0.6	Cows	20.2	46.3	43.5	13.9	46.7
	12. Polyurethane coated	0.6		22.8	51.0	44.6	18.5	51.2

a Supplementation of 30 % control release in forages. DMI = dry matter intage; DM = dry matter; CP = crude protein; DF = ; OM = organic matter; DF = dry fiber.

## 7 Manufacturing of slow-release urea products

Because of their low cost, non-protein nitrogen (NPN) sources can be more interesting than plant proteins. Depending on their diet, bacteria can obtain between 40 and 95 % of their nitrogen from ammonia, although protozoa do not utilize ammonia. For the role of protozoa (flagellates and mostly ciliates) in ruminants' nitrogen metabolism, see e.g. Ref. [2].

Urea is the most commonly utilized source of NPN, and inside the rumen, it is rapidly hydrolyzed to ammonia. Each kilogram of urea provides the equivalent of more than 5–6 kg of plant protein nitrogen supplement. Nitrogen urea is equivalent to 281 % of crude protein, while it is about 44 % and 50 % in cottonseed and soybean meals. Unwillingness by farmers to provide urea as the fodder for lactating ruminants may be rooted in its possible effects on food intake and fertility. This may lead to cases of death by toxicity if not adequately mixed with other diet components. Hence the rate of nitrogen release must be controlled, and several developments were made. Rumen ammonia accumulation can be reduced by increasing the amount of microbes that consume it. The use of microbial urease inhibitors was tested to achieve this goal, which had different results. An alternative solution to using urea in the diet is using slow-release urea (SRU) to regulate ammonia release more accurately with a carbohydrate digestion pattern [41]. The first slow-release urea product was biuret which consists of the bond of two urea molecules. The use of biuret in ruminant nutrition was evaluated during the 1970s [42].

The commercial success of these slow-release urea sources is determined by their cost compared to urea sources, conventional plant proteins, and their impact on microbial growth and the function of an animal. In addition, maximizing the use of a slow-release nitrogen source, such as biuret, takes time to get used to. The duration of this acclimatization period is dependent on the starch and protein in the diet [4]. It can last up to 42 days [4], so the administration of biuret is not feasible under any feeding conditions. Other urea protection methods may fix the compound so strongly that it loses availability for ruminal hydrolysis, while others were found to exert little effect on lowering the rate of intra-ruminal urea release [4]. Huston et al. [36] developed a slow-release urea pellet containing a mixture of urea, cornstarch, and carboxy resin. In vitro tests showed that the urea release rate decreased with increasing resin content. Ørskov et al. [43] developed a method involving urea in whole grains. Saturation is another method that leads to more efficient urea consumption. This process causes the whole grain to absorb urea. 50 % urea solution (wt.%) is sprayed on the whole grain while mixing in a vertical mixer to add 2 % (by weight) urea finally, no urea crystals are formed, and the grain completely absorbs urea. Bartley and Deyoe [9] developed Starea^1^ to improve ruminant urea uptake by ruminants. Starea is produced by thoroughly mixing grains (corn, wheat, sorghum, etc.) or other sources of commercial starch with an NPN source such as urea. Starea provides the energy available to ruminal microorganisms at the same rate as urea in such a way that the main compounds for synthesizing microbial protein are provided simultaneously.

Fishwick [44] developed a product with a coating of urea granules with sulfur and wax and studied nitrogen consumption by ruminants. Two sulfur-coated urea products were used. The first product contains 324 g of nitrogen per kilogram, and the coverage was 277 g of sulfur per kilogram, and the second product contains 342 g of nitrogen per kilogram and is covered with 236 g of sulfur per kg. Both products were coated with 20 g of wax per kg.

Males et al. [45] investigated the nutritional effect of slow-release molasses urea liquid products on dairy cows. Slow-release molasses urea reduced ruminal ammonia levels. Lower nitrogen digestibility for the SRU product indicates that the urea bond was powerful; therefore, not all urea is released into the rumen. Galo et al. [46] produced a controlled-release urea product called Optigen 1200, which was coated with a polymer that was compared to crude urea, and it was believed that the rate of ammonia release to microbes would decrease. Edwards et al. [47] showed that polymer-coated urea (Optigen 1200) reduced ruminal ammonia concentrations compared to crude urea [20]. In vitro experiments showed that the composition of calcium sulfate urea, in comparison with urea, decreased ruminal ammonia concentration; on the other hand, the population of cellulite bacteria (*Streptococcus* and *Staphylococcus*) increased. Many of these products have no advantage over urea because most of the nitrogen produced by them passes through the rumen intact and is not available to microbes [46]. Although many methods of slowing the release can effectively prevent ammonia poisoning, they rarely improve dietary nitrogen intake or affect livestock performance compared to conventional feed sources [48]. In fact, the main goal and challenge is to provide a product that controls the rate of ammonia release from urea without affecting its decomposition rate [45]. Bioplastic materials, such as polyhydroxyalkanoates (PHA), could be interesting coating materials for slow-release formulations. In any case, non-degradable polymers are to be avoided to prevent microplatic formation.

## 8 Nutritional effects of slow-release urea

SRU is usually compared to conventional oral urea to evaluate its effectiveness. In experiments based on comparing SRU with accurate protein sources, the presence of factors such as amino acids profile and available (fermentable) energy can mask the study of ruminal nitrogen consumption. Ruminal nitrogen availability affects food intake and digestibility [43]. Cherdthong et al. [49] reported that slow urea did not alter the release of total volatile fatty acids (VFAs), while leading to an increase in the proportion of propionate produced. Other researchers reported that comparing urea and slow-release urea showed no change in overall VFA and its ratios [33,40]. The mechanism of action of slow release causes the simultaneous urea release of energy and nitrogen in the rumen; as a result, there is an increase in the conversion of ruminal nitrogen to microbial protein [50]. Oldham [51] also suggested an increase in dry matter intake in diets containing soybean meal compared to urea to improve the supply of amino acids from soybean meal. However, further studies are needed to evaluate the animals' performance in justifying how slow-release urea affects the animals' consumption and weight gain patterns. The possibility of meeting the total requirements for metabolizable protein in beef cattle from the source of microbial protein produced in the rumen [52] may explain the lack of effect of slow-release urea in the diet on the apparent digestibility of crude protein [53]. Cherdthong et al. [49] reported that using slow-release urea increased total milk and fat yields compared to urea. Xin et al. [40] showed that even though milk production did not change with slow urea consumption, milk urea nitrogen was reduced compared to urea-containing diets. Use of lipid-coated slow-release urea in the final ration of Angus calves with flaked corn-based diets, Broderick et al. [54] did not yield significant differences in carcass performance or composition, while the ratio of overweight to dietary intake of the urea-containing diet with the same nitrogen tended to increase. Conversely, Taylor-Edwards et al. [33] found that urea-containing treatments had a tendency to gain more weight in beef than SRU, and they also showed that there were interactions with urea intake in the diet, as small amounts of urea supplementation led to better growth while SRU improved at higher dosage; therefore, the response to slow-release urea supplementation in replacement with oral urea was conditional and depending on the product of SRU, diet composition, and animals’ breeding conditions varied significantly.

## 9 The effect of using of slow-release urea

Digestibility and feed intake are common criteria for evaluating feed, to the extent that today the digestibility of feed or diet is a better qualitative indicator of its chemical value [55]. Puga et al. [38] found that dietary nitrogen with a ratio of 70:30 forage to slow-release urea significantly increased dry matter intake compared to the control group (100 % forage). They suggested higher digestibility due to higher ruminal fiber fermentation activity. Galina et al. [39] reported that an increase of 1.8 kg in dry matter urea supplementation per day showed that the digestibility of high fiber forage increases with the addition of ammonia and urea [43]. Replacing protein sources with Optigen had the same results.

Interestingly, in a study by Santiago et al. [56], when Optigen was used to replace soybean meal and rapeseed meal, the digestibility of fibers also increased, although ammonia nitrogen was high in both treatments. Reducing intake is possible by adding urea to the diet because of its bitter taste or physiological mechanisms; these include increased ammonia concentrations in the rumen and blood [57]. Elevated levels of urea, more than 20 g/kg of dry matter in the diet, maybe a reason to reduce DMI in urea treatment. However, this reduction in feed intake is due to the bitter taste caused by the addition of urea [58]; when urea is fed, excess nitrogen is not used in the rumen and is excreted. Slow-release urea and other similar compounds achieve a slower rate of nitrogen release in the rumen, allowing nitrogen to be used more efficiently in the rumen, and preventing ammonia toxicity [59]. Kertz [60] studied the dairy cows' diet. He added more than one percent urea to the diet without having any negative impact on the intake of feed. These findings are in line with a report by Neal et al. [61], which used slow-release urea at 0.49 %, and feed intake was not significantly changed. Allen reported in 2000 that when ruminal degradation of starch is increased as a percentage of dry matter, the DMI of dairy cows is reduced, and its specific mechanism may include hypertonic effects in the rumen and absorption of propionate in the liver, which may be due to reduced feed intake because of increased starch content and increased starch degradability. It may be that the uptake of feed metabolites is different, or that the ruminal osmolality is different, or that the blood availability of feed metabolites is different, or that the animals and diet have different response thresholds [62].

## 10 The effect of slow-release urea on ruminal fermentation parameters

Slow-release urea can continuously provide the ammonia needed for microbial growth for bacteria; it always gives at least 15–30 mg of nitrogen per deciliter for maximum microbial growth [7]. Strategies to improve the intake of such foods show that supplementation can correct nutrient imbalances for ruminal bacteria [63]. Slow-release urea products reduce ammonia concentrations by inhibiting the overproduction of ammonia-producing bacteria (HAP = hyper-ammonia producing). However, in vitro testing plays a significant role in protein breakdown due to the inability of protozoa to use ammonia; returning swallowed insoluble protein to ruminal fluid as a soluble protein [64]. This is one of the main reasons for the decrease in intra-ruminal ammonia concentration. Xin et al. [40] investigated the effects of polyurethane-coated urea on the concentration of VFA in a corn-based diet on dairy cows. Three experimental diets with the same nitrogen content (13 %) included oral urea, polyurethane-coated urea, and diets containing soybean meal; there was no important difference in total VFA between treatments. A similar concentration of total VFA showed no harmful effect of supplements on fermentation, and this may be because VFA formation is primarily affected by carbohydrate fermentation [65]. Table 2 highlights the impact of several SRU products on the fermentation process in the rumen.

**Table 2:** **Effect of slow-release urea (SRU) product on rumen fermentation parameters.** Reproduced from Andrieu and Wilde [4].

Source	Type of SRU	Suppl., diet (%)	Animal	NH_3_–N, (mg%)	Total VFA (mM/L)	VFA (%) –		
						C2	C3	C4
Galina et al. [39]	SRU	0	Beef	6.8	–	78.2	14.4	7.4
	SRU	1.8		12.3	–	72.2	16.0	11.8
Golombeski et al. [66]	Ruma Pro	0	Cows	5.4	54.0	62.9	21.2	11.4
	Ruma Pro	0.61		6.0	50.0	63.2	21.5	11.1
Taylor-Edwards et al. [33]	Urea	1.6	Steers	14.1	99.7	62.7	19.7	14.0
	SRU	1.6		8.9	103.2	63.6	20.3	13.8
Pinos-Rodríguez et al. [41]	Optigen®	0.6	Steers	–	97.6	52.0	34.9	13.0
	Optigen®	1.1		–	94.8	52.3	35.2	12.5
Xin et al. [40]	Urea	0.6	Cows	2.0	64.08	56.8	33.3	5.3
	Polyurethane coated urea	0.6		1.4	66.08	56.3	34.4	5.3

SRU = slow-release urea; VFA = volatile fatty acids.

## 11 The impact of SRU (slow-release urea) on rumen microbiome and microbial protein synthesis

Our goal in the rumen is to maximize the growth of microorganisms, and to convert RDP (rumen degradable protein) into microbial cells; on the other hand, it reduces nitrogen losses. Maximizing the use of degradable nitrogen not only improves amino acid stores in the small intestine, but also reduces nitrogen loss. Understanding the nitrogen compounds that are needed for rumen bacteria is essential for elucidating protein nutrition in ruminants and factors influencing fermentation in ruminants, especially fiber digestion. It is traditionally believed that cellulosic bacteria (cellulolytic ruminal bacteria) deploy ammonia as the only source of nitrogen. The results of some studies do not confirm this belief; however, Brito and Broderick [67] briefly stated the needs of ruminal bacteria, and he concluded that cellulosic bacteria use only ammonia as a source of nitrogen for growth. Without ammonia, they cannot grow with other nitrogen sources [56]. Stimulation of cellulosic species with different nitrogen sources showed that their optimal growth depended on the rate of ammonia release. In addition, according to experiments, the slow release of ammonia stimulates microbial growth and increases fiber digestion; microbial protein synthesis is more efficient than RDP derived from natural protein. Slow-release urea, compared to unprotected urea with a slow release of ammonia, is used less effectively by microorganisms.

## 12 The effect of slow-release urea (SRU) on milk yield and composition

Mazinani et al. [5] showed that in dairy cows fed with dry and liquid supplements containing urea (liquid supplement made from urea, minerals, and molasses), there was no impact on feed intake. Neither were milk yield nor fat content of the milk was affected. Naz and Sulaiman [68] reported that extrusion improved the optimal consumption of urea in diets containing high forage with starch compared to urea granules. The results showed that cows receiving cereal rations containing soybean meal and Starea consumed more cereals and consumed more milk than diets containing urea. Tedeschi et al. [18] used a diet containing polymer-coated slow-release urea (Optigen 1200) and observed that feed efficiency improved, which was not unexpected because cows consumed less dry matter, and slow-release urea consumption did not reduce daily milk yield. Optigen 1200 positively affected fat milk yield and increased milk protein yield; however, adding coated urea had no notable influence on milk composition. Galo et al. [46] reported that feeding high-yielding dairy cows during lactation by replacing a combination of urea and protein sources with diets containing 0.77 % polymer-coated urea had no effect on milk production; experimental diets also did not affect milk fat and protein percentage. The milk fat percentage was about 3.7 %, indicating ruminal fermentation and proper fiber digestion. Inostroza et al. [69] experimented to determine the effect of Optigen as a source of NPN on milk yield, composition, and addition efficiency. They reported that milk yield increased with limited replacement of Optigen with urea granules and oilseed meal in the diets of dairy cows, milk fat and protein yields were not affected by the treatments. Highstreet et al. [34] tested a slow-release encapsulated urea product (Nitroshure) according to the manufacturer containing 0.9 units of urea and 0.1 units of fat. The use of SRU in diets with a high content of insoluble nitrogen increased early in lactation in high-yielding lactating cows compared to the average amount of urea equivalent in terms of nitrogen, milk fat, protein, and output energy, while in the middle of lactation, it had a limited effect. Xin et al. [40] found no effect on milk fat, lactose content, milk production, and energy-modified milk production regardless of milk protein content; significant differences were observed between experimental diets. Cows fed urea diets with a polymer coating and soybean meal had similar milk protein concentrations; both diets were higher than diets containing dietary urea. The percentage and yield of milk protein for cows receiving polymer-coated urea rations were higher than oral urea rations and were like soybean meal rations. The animals’ ability to metabolize excess nitrogen released in the rumen is one of the many factors on which the advantages of SRU depend, and future studies need to quantify the effect of these sources, especially in the diet of high-yielding dairy cows. Most prior studies have focused on feeding low-yielding animals with high-fiber diets (Table 3).

**Table 3:** **Impact of feeding slow-release urea on milk production in dairy cows. Source:** Cherdthong and Wanapat [20].

Source	Type of SRU	Suppl. Diet (%)	Animal	Milk Yield (kg/d)	Milk composition (%) –		
					Fat	Protein	Lactose
Galo et al. [46]	Urea	0.3	Cows	35.8	3.8	3.1	–
	15. Optigen®	0.816.		34.8	3.6	3.1	–
Golombeski et al. [66]	Ruma Pro	0	Cows	26.1	4.2	3.7	4.8
	17. Ruma Pro	0.6118.		26.2	4.4	3.7	4.8
Inostroza et al. [69]	Optigen®	0	Cows	35.4	3.7	3.0	–
	19. Optigen®	114^a^20.		35.9	3.7	3.0	–
Highstreet et al. [34]	Urea	1.8	Cows	46.9	3.6	2.8	4.7
	Encapsulated urea	1.721.		47.6	3.7	2.8	4.7
Xin et al. [40]	Urea	0.6	Cows	32.48	3.71	2.94	5.09
	22. Polyurethane coated urea	0.623.		34.53	4.01	3.16	4.99

a Fed 114g of Optigen® per head per day. SRU = slow release urea.

## 13 Single cell protein (SCP) as potential alternative non-conventional nitrogen source

In aquaculture, fishmeal is a common protein source, as well as soybean meal, where several concerns over sustainability exist. Single cell protein (SCP) has been suggested as a potential replacement [70], also for chicken feeding and other monogastric animals [71,72]. SCP can be made from waste biomass [73]. For feed and food applications, there are bacterial, fungal, and algal SCPs. For an overview of sources for bacterial SCP, see Table 4.

**Table 4:** Bacterial single cell protein from different substrates. Source: Raziq et al. [74].

Bacteria	Substrate	Protein content (%)
*Haloarcula* sp. *IRUI*	Petrochemical wastewater	76
*Methylococcus capsulatus*, *Ralstonia* sp., *Brevibacillus agri*, *Aneurinibacillus* sp.	Methane (Natural gas)	67–73
*Bacillus subtilis*	Ram horn	71
*Methylomonas* sp.	Methane salt broth	69
*Bacillus cereus*	Ram horn	68
*Escherichia coli*	Ram horn	66
*Rhodopseudomonas palustris*	Latex rubber sheet wastewater	55–65
*Corynebacterium ammoniagenes*	Glucose + Fructose	61
*Rhizospheric diazotrophs (whole microbial community)*	Brewery wastewater	>55
*Bacillus pumilis*	Potato starch processing waste	46
*Cupriavidus necator*	Synthetic growth medium	40–46
*Bacillus licheniformis*	Potato starch processing waste	38
*Bacillus subtilis*	Soybean hull	12

According to the EU feed catalog 68/2013, several yeasts and bacterial single cell proteins are approved for use. For calves, up to 8 % bacterial SCP was approved in the EU in 1995 [75].

The use of SCP for cattle is discussed in Anupama and Ravindra [76]. They summarize the use of algae in cattle breeding, i.e. *Ascophylluem, Fucus, Laminaria* (Japan, USA, New Zealand), *Pelvwtia* (Norway, France, USA, Denmark, New Zealand) and *Rhodymenia* (France). Van Weerden and Huisman [77] studied digestibility of SCP with veal calves. They found that the average digestibility of the crude protein in ‘Pruteen’ was 93.6 %, which came out almost as high as milk protein (94.2 %).

Urea and single-cell protein were investigated as feed supplements among fattening calves by AIwasn et al. [78]. Suman et al. [79] studied SCP for calf fattening.Thayer [80] suggested the production of SCP for cattle feed by bacteria from mesquite wood.

SCP based on yeast (Candida utilis) was studied as a cattle feed supplement by Nigam [81]. SCP can be particularly interesting in case of a global food catastrophe as described by García Martínez et al. [82]. It is possible for nitrogen to come from the air, ammonium, waste streams, or N-fixing bacteria. A recent review in SCP for feed and food was published by Li et al. [83].

## 14 Conclusion

For ruminants to produce meat and milk, large quantities of nitrogen must be available, which has detrimental effects on the environment. Feed costs are reduced and productivity is maintained by finding solutions. Compared to regular urea, slow-release urea lowers blood urea nitrogen levels, along with slow-release urea and other similar products with a reduced nitrogen release rate in the rumen, uses nitrogen more efficiently to prevent ammonia poisoning and increases nitrogen retention effectively. To decrease diet expenses and to provide the required nitrogen deficit without reducing digestibility, low(er) quality fodder may be deployed in the diet to provide the nitrogen deficit without reducing digestibility, utilize nitrogen retention, and increase fiber digestion based on the results obtained in the absence of high-priced fodder. Finally, it is concluded that slow release urea can be used in feeding ruminants without any side effects.

## Data availability statement

Since this is a review article, no new data were generated.

## CRediT authorship contribution statement

**Masoumeh Niazifar:** Writing – original draft. **Maghsoud Besharati:** Writing – original draft. **Muhammad Jabbar:** Investigation, Formal analysis. **Shakira Ghazanfar:** Investigation, Formal analysis. **Muhammad Asad:** Visualization, Resources, Methodology, Investigation. **Valiollah Palangi:** Writing – original draft, Conceptualization. **Hüseyin Eseceli:** Project administration, Methodology. **Maximilian Lackner:** Writing – review & editing.

## Declaration of competing interest

The authors declare that they have no known competing financial interests or personal relationships that could have appeared to influence the work reported in this paper.

## Footnotes

1 This is an intimate mixture of gelatinized starch (from wheat) with urea.
